# Irritability of the Bladder1Read at the 84th semiannual meeting of the Medical Society of the County of Erie, June 13,1905.

**Published:** 1905-09

**Authors:** J. Henry Dowd

**Affiliations:** Buffalo, N. Y.; Genitourinary Surgeon to the Mercy Hospital, 354 Franklin Street


### BUFFALO MEDICAL JOURNAL.

VOL. LXI. SEPTEMBER, 1905. No. 2

### ORIGINAL COMMUNICATIONS.

### Irritability of the Bladder.[1]

By J. HENRY DOWD, M. D., Buffalo, N. Y.
Genitourinary Surgeon to the Mercy Hospital.

IRRITABILITY of the bladder is a condition in which this viscus is incapable of retaining its contents for a definite time without undue exertion on the part of the individual. A definite time varies according to the amount of urine secreted. Thus, there may be absolutely no disease of the vesical mucous membrane yet, as in diabetes or hysterical conditions, evacuation may take place every hour. One must understand the physiology of urinating before an estimate can be put upon normal evacuation.

In the male, urine is held in the bladder by the action of two muscles, the compressor and the internal sphincter; the latter at the urethre vesical opening, the former about one and one-half inches anterior thereto. As the bladder becomes filled, at a certain time the internal sphincter, which consists of only a few muscular fibers, gives away, thus allowing the urine to enter the posterior urethra as far as the compressor. It is now seen that the posterior urethra forms what may be described as the neck of the bladder. From most careful observation the writer has found that this phenomena takes place from one and one-half to two hours after a previous evacuation in an individual secreting the normal amount, that is, about 50 ounces of urine per diem.

It may be stated that with the normal amount of urine the bladder should retain its contents for from six to eight hours without the sensation of a desire to urinate being evinced. In other words the bladder should be able to contain at least eight or ten ounces of urine without any distress whatever. Of course

1. Read at the 84th semiannual meeting of the Medical Society of the County of Erie, June 13, 1905.

its capacity can be greatly increased with little or no inconvenience, but a good rule would be to empty the bladder, on an average, five or six times during waking hours.

Bearing in mind the fact, and it should be considered such, that the posterior urethra is a part of the urinary bladder, it is evident that trouble in this region will, and always does, produce frequency of urination, yet there may be no trouble inside the sphincter muscle. An example may be cited in posterior urethritis, acute or chronic. Eliminating abnormal secretion, as in diabetes and conditions of the general nervous system, the causes of irritability of the bladder may be grouped as follows: inflammatory, congestive, and neurotic. To these may be added reflex conditions, but here we will find that there is some pathological condition, even though it be trivial in nature, somewhere along the urinary tract, for example, long prepuce, any irritation of the glans penis, and the same condition around the vulva. Inflammation which has involved the urinary tract may have resolved, yet frequency may continue to be pronounced. Examples of such may be vesiculitis or follicular prostitis. A few of the most important causes of irritability due to inflammation, and in order of frequency are:

Acute or chronic posterior urethritis.

Acute or chronic follicular or parenchymatous prostatis.

Cystitis.

Vesiculitis, acute or chronic.

Congestive conditions.

The bladder may be congested for a brief period due to irritating urine, but as such a condition is usually temporary, reference will only be made to these when they are more or less persistent. The most frequent condition in this class we find in the female, where every time she hears water running or puts her hands in water the desire to urinate is provoked. This is due to a congestion at the urethrovesical opening, plus a neurotic state.

*Neuroses.*—These may be either local or general. One of the most important and sensitive portions of the whole nervous system is situated in this region, and any interference with it will be shown by action on the bladder. As to the general nervous system as a factor no better example can be cited, than when, from sudden fright, a person will empty his bladder several times in the course of an hour.

*Symptoms.*—The symptoms of irritable bladder are so well known that it would seem a waste of time to repeat them here. Suffice it to say, they will usually be evident according to the cause present. Thus, we may have frequency with absolutely no pain whatever; on the other hand, pain may be one of the constant and aggravating symptoms. Epitomised, symptoms of irritable bladder may be said to be, frequency of urination, painful or not, with pain deeply situate in the region of the symphysis pubes, rectum or perineum.

*Treatment.*—The treatment of irritable bladder, as the treatment of other conditions, depends entirely upon the discovery of the cause and the removal of the same where possible. Medication either internal or external should not be thought of until the urine has been subjected to analysis. By this we will be enabled to discover diabetes, Bright's disease, hysteria, lithemia, and other conditions which may cause a frequent emptying of the bladder, yet there may be no disease of the viscus. Inflammation will also be recognised, for, if pus is present in the fluid, it indicates inflammation somewhere along the tract. When this is discovered its source must be sought and removed, if we hope to bring about a normal condition.

Time at disposal will scarcely allow the taking up minutely of each condition, therefore a few general rules must suffice. In the majority of cases nervous influences are marked, and no matter what may be the cause, it is well to incorporate in every prescription drugs known to have special influence on this region. If there is considerable pain an opiate should be included. The writer has found the following to be most serviceable—ext. hyoscyami gr. j—ext. stamonii gr. ¼ to ½; one pill every three to five hours. If there is great pain, morphine in small doses can be added. In lithemic conditions where crystals appear, this condition must be attended to according to the cause thereof. Thus, if due to a diet rich in lime, and the like, this should be regulated. Where it may be due to malassimilation, the digestive tract needs attention.

At times the general nervous system needs care. Thus, I have seen a case where the urine was not materially increased in quantity; there was absolutely no disease of any part, yet the woman was urinating say every fifteen minutes to two hours day and night. In these cases rest and sedatives are all important. In irritable conditions where hypertrophied prostate is the predisposing and infection with foul urine the exciting cause, no drug with which I am familiar will give relief as certainly as uriseptin. It is the writer's practice when using this drug for any purpose, to add to it either ext. of hyoscyamus or stramonium.

In tubercular conditions but little can be accomplished. Washing out the bladder is contraindicated, excepting where a simple inflammation may be engrafted upon the tubercular process, due to the use of dirty catheters. Cutting operations in this condition should never be thought of, except as a last resort, and then only with a view to enthanasia. Stone, foreign bodies, and the like, should be removed at once, the bladder being cared for until resolution has been brought about. Excepting in acute cystitis, all local medications by solutions to the mucous membrane behind the compressor should be avoided, until chronicity has been established, when, if resolution seems to be at a standstill, local medication may be used.

Briefly speaking, these conditions may be dealt with as follows:

In acute posterior urethritis—sedatives, when required.

In chronic posterior urethritis (not before the fifteenth day) irrigations of silver nitrate 1-16000, 1-12000, 1-10000 respectively daily until recovery.

In acute vesiculitis—sedatives only, preferably per rectum.

In chronic vesiculitis—stripping of the vesicles every fourth day, using a suppository of ichythol, belladonna and hyoscymus at night. At times the psychrophore, using hot water, 125° F., will be of great advantage.

In acute parenchymatous or follicular prostatitis—leeches, at least six, applied to the perineum.

In the chronic form of the same condition—sounds, irrigations and suppositories.

In acute cystitis we may expect the most rapid results. One irrigation of silver nitrate 1-16000 applied from the meatus will bring about resolution in a few hours, providing the diagnosis is correct. Chronic cystitis depends entirely upon the cause, which must be sought and removed, after which irrigation of silver nitrate will usually give good results.

A condition of irritability, at times a very distressing one, is found in women. This is not due to inflammation, but to congestion. The most prominent symptom of this, as already alluded to, the desire to urinate on hearing running water. For this condition nothing will be more effective than ext. stramonium and tr. cantharidis, the latter in drop doses, three times a day.

*Summary.*—1. Search for a cause in every case, and remove it if possible.

2. Examine the urine in every case; if a possible cause is found, use appropriate remedies.

3. Consider the nervous system, either local or general, a predisposing cause, in every case using sedatives for its control.

4. Never use any local medication in acute inflammatory conditions, except in acute cystitis.

354 Franklin Street.

